# Protocol for studying parasitoid-induced long-term effects in *Drosophila*

**DOI:** 10.1016/j.xpro.2024.103438

**Published:** 2024-11-04

**Authors:** Madhumala K. Sadanandappa, Giovanni Bosco

**Affiliations:** 1 Department of Molecular and Systems Biology, Geisel School of Medicine at Dartmouth, Hanover, NH 03755, USA

**Keywords:** model organisms, behavior, evolutionary biology

## Abstract

*Drosophila* and its parasitoids provide an ecologically relevant model for studying host-parasitoid biology, focusing on the behavioral and physiological responses involved in host defensive strategies and parasitoid countermeasures. Here, we outline a protocol for rearing *Pachycrepoideus*, a pupal parasitoid wasp, and a behavioral assay to assess the long-term impact of parasitoid exposure on adult *Drosophila*. We detail the steps for preparing and cohabiting *Drosophila* with the wasps, documenting egg-laying, and analyzing reproductive responses and eclosion in fruit flies.

For complete details on the use and execution of this protocol, please refer to Sadanandappa et al.^1^

## Highlights

• Instructions for husbandry and culturing of *Pachycrepoideus* in the laboratory
• Steps for studying parasitoid-induced reproductive responses in *Drosophila*
• Protocol adaptability across *Drosophila* parasitoid wasp and its host species

## Abstract (editor highlights)

Publisher’s note: Undertaking any experimental protocol requires adherence to local institutional guidelines for laboratory safety and ethics.

## Graphical abstract

**Figure:**
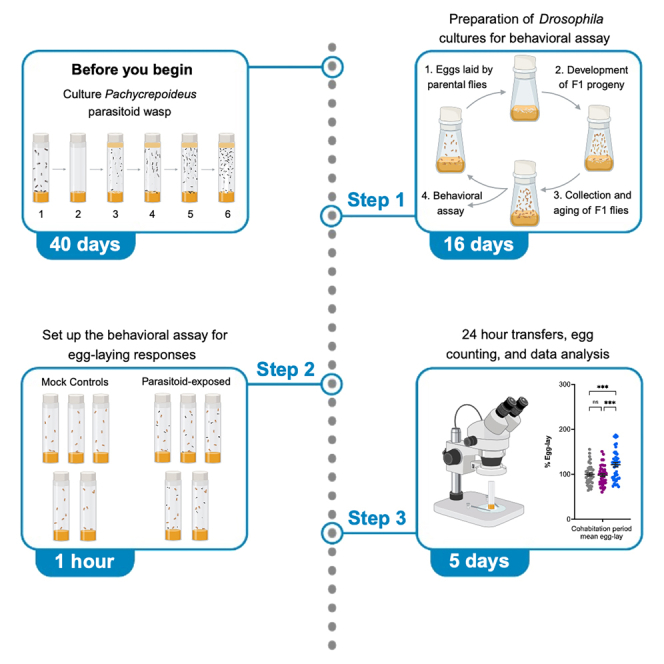

## Before you begin

The following protocol provides step-by-step procedures for testing and analyzing the egg-laying responses of wild-type adult *Drosophila melanogaster* females (Canton S or CS) to *Pachycrepoideus* (PP), a pupal parasitoid wasp. However, we have also used this assay to test other parasitoid wasp species, such as *Leptopilinia* (larval parasitoid) and *Trichopria* (pupal parasitoid). Importantly, these steps apply to *Drosophila* of different genotypes, strains, and species.

### Preparation of *D.* *virilis* cultures

**Timing: 2 days**

1. Prior to beginning, prepare standard *Drosophila* culture medium using following reagents (Table 1).
  a. Add the above mixture to the warm water and bring to boil by stirring.
  b. Let the medium cool to 60°C before adding propionic acid and NIPAGIN.
  c. Immediately dispense food into narrow vials or plastic bottles.
  d. Cover the freshly poured fly food vials or bottles with a sterile cheesecloth and allow the food to solidify overnight at room temperature (RT - 22°C–25°C) prior to plugging.
  e. Store the fly food at 18°C for a week or 4°C for up to a month.

**Table 1:** Cornmeal molasses yeast medium for fly culturing

Reagent	Final concentration (%)	Amount
Agar	1%	10 g
Molasses	7.6%	76 g
Cornmeal	7.6%	76 g
Yeast	5%	5 g
Propionic acid	0.2%	2 mL
NIPAGIN	0.4%	4 mL
ddH_2_O	N/A	to 1 L
**Total**	**N/A**	**1 L**

**CRITICAL:** Before introducing flies or wasps into the food vials and bottles, ensure the fly medium is at RT. Use Kimwipes to remove condensed water, if necessary.

**Figure 1:**
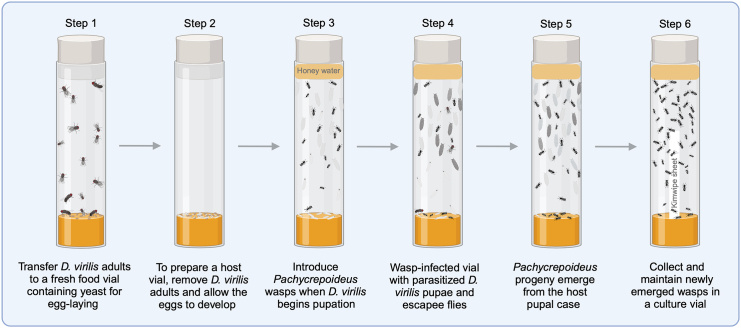
*Pachycrepoideus* wasp culturing The schematic illustrates the workflow for rearing *Pachycrepoideus* parasitoids wasp on *D. virilis* pupae in the laboratory setting.

2. Use carbon dioxide (CO_2_) to anesthetize a bottle of *D. virilis* and place them on a fly pad.
  a. Add approximately 20 active yeast granules into a fresh fly food vial.
  b. Using a paintbrush (round size 4/0 or 3/0), transfer ten female and five male *D. virilis* (parental flies) into the vials and leave them on their side until the flies recover from CO_2_ anesthesia.
    ***Note:****D. virilis* males are relatively smaller than female flies. Male flies have bright red gonads visible through the cuticle on the ventral side of the abdomen.
  c. Allow parental flies to lay eggs in a food vial for 4–5 days at RT (Figure 1).
    ***Note:*** For optimal egg-laying, use 3 to 20-day-old-mated *D. virilis* females.

### Preparation of *D.* *virilis* host vials for *Pachycrepoideus* infection

**Timing: 15 min**

3. After laying eggs, either transfer *D. virilis* parental flies to a new food vial with yeast granules or discard them into a morgue.

***Note:*** Check the vials for signs of larvae. If the culture media is dry, add a few drops of distilled water. Discard any vials with fewer than 50 eggs or if they show signs of mold or fungal growth.

4. Allow the F1 offsprings to pupate at RT (Figure 1).

***Note:*** Due to its larger body size, *D. virilis* life cycle is longer than that of *D. melanogaster* (9–10 days at 25°C).

### Preparation of *D. melanogaster* cultures for behavioral assay

**Timing: 9–10 days**

5. Use CO_2_ to anesthetize CS flies, a wild-type strain of *D. melanogaster*, cultured on a standard fly medium under laboratory conditions.

***Note:*** The laboratory conditions refer to an incubator with 25°C, ∼60% relative humidity, and 12:12 h light/dark cycles, unless otherwise stated.

***Note:*** Use 3 to 15-day-old CS flies and avoid collecting flies from overcrowded, mite-infested, or fungal-contaminated bottles.

6. After anesthetizing, separate male and female CS flies on a fly pad.
7. Place 25 female and 10 male CS parental flies into a bottle containing a fresh medium. After flies recover from anesthesia, move the bottles to the incubator.

***Note:*** Male CS flies are generally smaller than females and have a thick, darker abdominal band and a sex comb on each of their first pair of thoracic legs or forelegs.

**CRITICAL:** To prevent flies from sticking to the media, ensure the bottles are free of condensed water before placing the flies inside the bottle. After transferring the flies, leave the bottle on its side until the flies recover from CO_2_ exposure (typically 1–2 min).

8. After three days of egg-laying (which will develop into F1 flies), transfer the adult parental CS flies to a new bottle to continue the cultures or discard them to a morgue.

***Note:*** Consider the age of the parental CS flies for the culture expansion. For example, if the parental flies are 3 to 5-day-old, transfer them to new bottles every three days for three transfers. If the parental flies are 6 to 8-day-old, transfer them to new bottles every three days for two transfers. We recommend using younger parental flies, with an age not exceeding 15 days.

***Note:*** We recommend visually inspecting the bottles for larval activity and discarding any bottles with poor egg-lay. Add a few drops of distilled water if the fly media is dry.

9. Continue maintaining the cultures in the incubator until the F1 CS adults emerge from the bottles. These CS flies are used for the egg-laying assay detailed below.

## Key resources table

**Table:** 

REAGENT or RESOURCE	SOURCE	IDENTIFIER
**Chemicals, peptides, and recombinant proteins**		

Gelidium agar	MoorAgar	Cat# 41706
Corn	MP Biomedicals	Cat# 0290141180
Molasses	Reinhart Foodservice	Cat# DW816
Yeast	Phileo by Lesaffre	Cat# 73050
Propionic acid	Fisher Scientific	Cat# A258-500
Methyl 4-hydroxybenzoate (NIPAGIN)	Sigma-Aldrich	Cat# H5501
Raw and unfiltered honey	Nature Nate’s Honey Co.	https://www.naturenates.com

**Experimental models: Organisms/strains**		

Canton S (CS)	Bloomington Drosophila Stock Center	N/A
*D. virilis*	Todd Schlenke, University of Arizona, Tucson	N/A
*Pachycrepoideus* sp.1	Todd Schlenke, University of Arizona, Tucson^2^	N/A
*Trichopria* sp.1	Todd Schlenke, University of Arizona, Tucson^2^	N/A

**Software and algorithms**		

Microsoft Excel	Microsoft	https://www.microsoft.com
GraphPad Prism 10.2.3	GraphPad Software	https://www.graphpad.com
BioRender	BioRender	https://biorender.com

**Other**		

Square bottom *Drosophila* bottle	Genesee Scientific	Cat# 32-130
Flugs plastic fly bottles	Genesee Scientific	Cat# 49-102
Narrow *Drosophila* vials	Genesee Scientific	Cat# 32-116SB
Flugs narrow plastic vials	Genesee Scientific	Cat# 49-102
Paint brush, round size 4/0 or 3/0	N/A	N/A
Fly pad, CO_2_ anesthetizing apparatus	Genesee Scientific	Cat# 59-119
Kimwipes	Kimberly-Clark Professional	Cat# 34155
Stereomicroscope	Zeiss	Stemi 2000
Incubator	Percival Scientific	DR41VL
Fiber optic light source	Fisher Scientific	Laxco PIFOS150IB

## Step-by-step method details

### Rearing *Pachycrepoideus* parasitoid wasps

**Timing: 1 month**

This step involves cultivating *Pachycrepoideus* parasitoid wasps on *D. virilis* pupae and maintaining the wasp cultures in the laboratory (Figure 1).

1. Prepare the vial containing *D. virilis* pre-pupal stages, which serve as the host for *Pachycrepoideus* parasitoids.
2. Add a 1:1 ratio of honey water on the inner side of the host vial plugs, which will act as food for *Pachycrepoideus* adults.
3. Use CO_2_ to anesthetize *Pachycrepoideus* adults (5–10-day-old) and place them on a fly pad.

***Note:*** Since *Pachycrepoideus* parasitoids are sensitive to CO_2_ anesthesia, use low pressure (approximately 10 PSI) and limit prolonged exposures.

**Figure 2:**
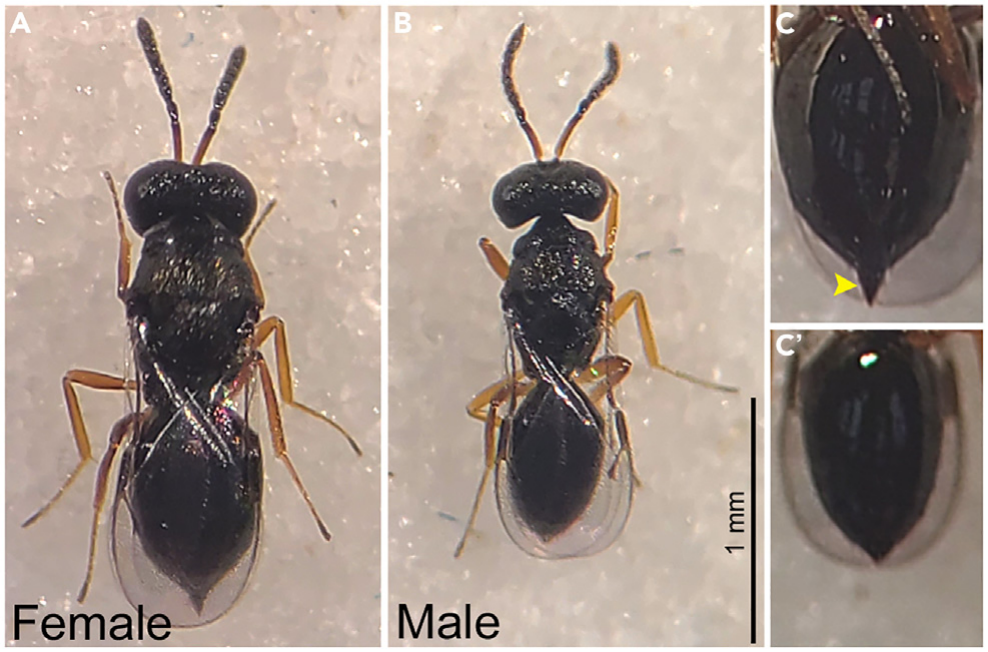
Photographs of *Pachycrepoideus* wasps The dorsal view of (A) female and (B) male *Pachycrepoideus* wasps with a scale bar of 1 mm. The posterior ventral abdominal view of (C) a female with the ovipositor (yellow arrowhead) and (C′) a male *Pachycrepoideus* wasp. Images adapted with permission from Sadanandappa et al.^1^

4. Identify and separate female and male wasps under a stereoscope.
  a. Female *Pachycrepoideus* wasps are relatively larger than males (Figures 2A and 2B).
  b. Female wasps have a specialized needle-like structure called an ovipositor at the abdominal tip, used to inject eggs into the host hemolymph (Figure 2C).
5. Using a paintbrush, carefully transfer 10 female and 5 male *Pachycrepoideus* adults to the food vial containing *D. virilis* early pupal stages and cover the vial with honey-supplemented plugs.

**CRITICAL:***Pachycrepoideus* reproduces through haplodiploidy, where male wasps are haploid, and females are diploid. Therefore, in addition to adding male wasps to the host vial, make sure mated females are used for parasitization to ensure the proportionate emergence of male and female wasp progeny.

***Note:*** For a better yield of wasps, we recommend introducing *Pachycrepoideus* wasps when host larvae initiate pupation. Early pupal stages of the host are more susceptible to parasitoid infection than later stages.

6. Leave the *Pachycrepoideus*-infected vials on their side until parasitoids recover from anesthesia, and ensure parasitoids are freely moving before turning the vial upright.

**CRITICAL:** Handle the wasps carefully and gently. They can quickly die from excess CO_2_, harsh handling, or sinking in wet food during anesthetization and transferring.

7. Maintain the wasp-infected vials at RT for parasitization of the host pupae and development of *Pachycrepoideus* parasitoids (Figure 1).

***Note:****Pachycrepoideus* parasitoids belong to the Pteromalidae family and attack *Drosophila* pupae to lay their eggs. The pre-imaginal stages of the wasps develop inside the host by feeding from within, and adult wasps emerge from the *Drosophila* pupal case.

***Note:*** Using the above-described steps, *Pachycrepoideus* cultures can also be maintained on *D. melanogaster*. Refer to Todd Schlenke’s laboratory resources.

8. Depending on the laboratory conditions, the *Pachycrepoideus* progeny emerge in the wasp-infected vials approximately 22–28 days post-infection.
9. Prepare a culture vial for maintaining adult *Pachycrepoideus* parasitoids (Figure 1).
  a. Add diluted honey to the plugs.
  b. Insert a folded Kimwipe sheet inside a vial containing fresh fly food to absorb excess moisture.
  c. CO_2_ anesthetize the newly emerged adult wasp, place them on a fly pad.
  d. Transfer approximately 50 adult wasps per culture vial and cover with honey-supplemented plugs.
  e. Leave the culture vials on their side until the wasp recovers from CO_2_ exposure and maintain the vials at RT.
  f. For a continuous collection of newly emerging wasps, repeat the above steps (a to e) every 3–4 days.
  g. To ensure the longevity of the adult wasps, shift them to a fresh culture vial every 4–5 days.
10. These *Pachycrepoideus* cultures can be used for the assays detailed below and for parasitizing a new host vial to maintain a continued supply of the wasp cultures in the laboratory.

### Egg-laying assay

**Timing: 11 days**

**Figure 3:**
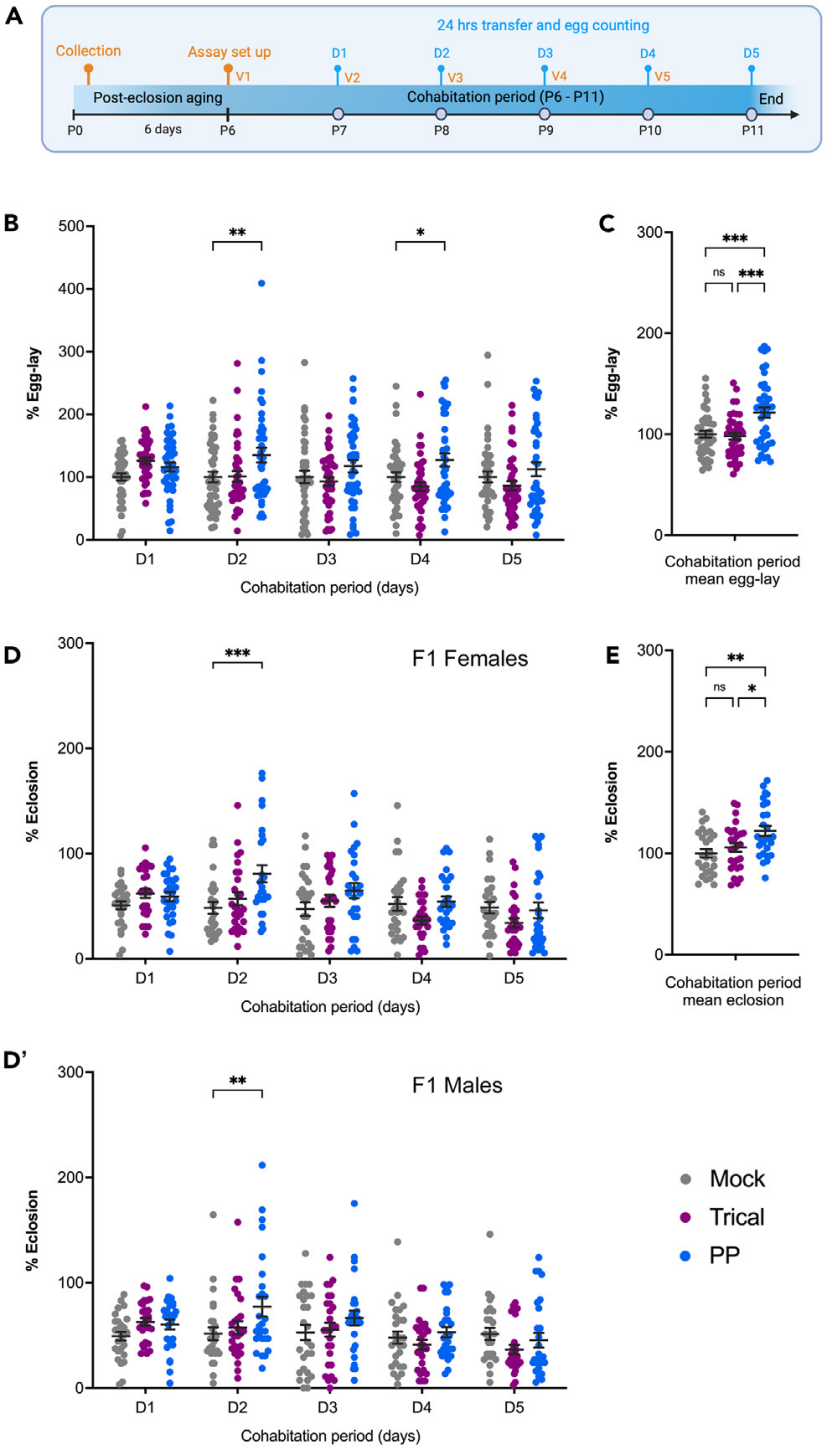
Assay for *Pachycrepoideus*-induced behavioral modification in *Drosophila* (A) Schematic outlining the workflow for examining the influence of *Pachycrepoideus* cohabitation on *Drosophila* egg-laying responses. On post-eclosion day 6 (P6), flies cohabitated with the parasitoid wasps. Every 24 h, all insects were transferred to a fresh vial (V1 to V5) for five days (D1 to D5), and the number of eggs laid was recorded. Unexposed controls that were not exposed to wasps were handled similarly. Dot plots showing (B) the 24 h and (C) 5 days mean egg-laying responses of CS flies exposed to pupal parasitoids – Trical (purple) (*n* = 42) and *Pachycrepoideus* wasp (blue) (*n* = 42) along with unexposed controls (gray) (*n* = 42). Dot plot showing (D) the 24 h and (E) 5 days mean F1 adult eclosion of CS exposed to pupal parasitoids – Trical (*n* = 27) and *Pachycrepoideus* wasp (*n* = 27) compared to unexposed controls (*n* = 27). The data represents the mean ± SEM from at least three independent experiments. Mock, Trical, and *Pachycrepoideus* wasp-exposed data were presented in gray, purple, and blue, respectively. ∗∗∗*p* < 0.001, ∗∗*p* < 0.01, ∗*p* < 0.05 and ns for non-significant (*p* > 0.05) determined by one-way ANOVA with Tukey’s multiple comparison tests for (C) and (E) and two-way ANOVA with Dunnett’s multiple comparison tests for (B) and (D). Quantitative egg-laying and eclosion data adapted with permission from Sadanandappa et al.^1^

To perform the behavioral assay for studying the egg-laying responses of CS females to *Pachycrepoideus* parasitoids, follow the steps below (Figure 3A).

11. Collection of age-matched wasps and flies for setting up the behavioral assay.
  a. For *Pachycrepoideus* parasitoid wasp:
    i. Remove all *Pachycrepoideus* progeny from the vials infected with the wasps (from step 8).
    ii. Allow 12 h before collecting newly emerged wasps from these vials to the collection vial, as described in step 9.
    iii. Age the wasps for 7 days at RT before using them for the behavioral assay.
  b. For CS flies:
    i. Discard all the F1 progeny eclosed in the CS culture bottles into a morgue and continue maintaining bottles in the incubators (See preparation of D. melanogaster cultures for behavioral assay).
    ii. Within 12 h of clearance, collect the newly emerged male and female CS flies into a fresh fly bottle (approximately 50 flies/bottle) without CO_2_ anesthetization.
    iii. Maintain the collected 0–12 h old CS flies in the incubator for 6 days (post eclosion day 6/P6) before setting up the assay.

**CRITICAL:** Avoid collecting F1 progenies from old, overcrowded, or contaminated vials/bottles.

12. Setting up the egg-laying assay for *D. melanogaster*.
  a. Setting up the *Pachycrepoideus*-exposed group (PP-exposed).
    i. CO_2_ anesthetize 7-day-old *Pachycrepoideus* wasps from the culture vial (step 11a.iii).
    ii. Separate male and female wasps on the fly pad (Figure 2).
    iii. Gently place five female *Pachycrepoideus* wasps in a fresh food vial.
    iv. Anesthetize 6-day-old CS flies (step 11b.iii) and transfer five female and two male flies into a vial (V1-PP-exposed) with the female *Pachycrepoideus* wasps.
  b. Setting up a control group (mock/unexposed).
    i. Repeat the above steps, but do not introduce *Pachycrepoideus* wasps to set up the unexposed control group (exclude steps 12a.i-iii).
    ii. Transfer five female and two male CS flies into a fresh food vial (V1-mock) without the wasps.

***Note:*** We recommend setting up at least five vials each for the unexposed control and PP-exposed group. These steps can also be used to test different *Drosophila* genotypes and parasitoid species.

**CRITICAL:** Thoroughly clean the fly station and the work area with 70% ethanol after handling parasitoid wasps. Additionally, avoid exposing *Drosophila* stocks to parasitoid wasps during transfers and while maintaining the cultures.

**CRITICAL:** CO_2_ impacts *Drosophila* oviposition behavior. Therefore, limit prolonged exposure to high concentrations of CO_2_ during the assay setup.

13. Place all egg-lay assay vials (V1) on their sides and allow the insects to recover from anesthesia before moving the vials to an incubator in the upright position.

**CRITICAL:** During anesthesia recovery, keep the control and PP-exposed vials away from each other. Place them on a separate fly rack on a different shelf of the incubator.

14. After 24 h of egg-laying, carefully transfer the control and PP-exposed groups into fresh food vials (V2) and return them to the incubator.

***Note:*** Handle insects carefully during transfers to prevent their escape, specifically CS flies. To avoid causing mechanical stress, tap the vials gently and/or use the negative geotaxis behavior of flies for transferring. Holding a fresh food vial opening directly over a vial with moving flies takes advantage of their natural propensity to crawl and fly upward.

15. Using a stereomicroscope, manually count the *Drosophila* eggs in each control and PP-exposed vial (V1). This data corresponds to 24 h of egg-laying on day 1 (D1).

***Note:****D. melanogaster* eggs are oval and about 0.5 mm long, with dorsal appendages on the anterior side. Adjust the external light source of the microscope for better visualization and contrast the eggs laid on food.

16. After counting the eggs, return all vials (V1) for both groups into the incubator to allow the F2 CS progenies to develop for performing the eclosion assay. If not, discard them.
17. Repeat the transfer and egg counting steps (14–16) every 24 h from days 2 through 5 (D2 to D5) to record the egg-laying responses of CS flies to mated female *Pachycrepoideus* wasps.
18. Analyze the data and plot graphs using Microsoft Excel or GraphPad Prism (Figure 3).

***Note:*** After five days of egg-lay analysis, the unexposed control and PP-exposed CS females can be used to analyze the ovaries as reported in our published manuscript.^1^

**Scheme 1:**
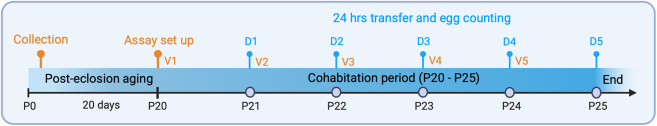
The workflow for examining the egg-laying responses of aged CS flies On post-eclosion day 20 (P20), flies cohabitated with the parasitoid wasps (P20 - P25). Every 24 h, all insects were transferred to a fresh vial (V1 to V5) for five days (D1 to D5), and the number of eggs laid was recorded.
  b. To investigate how CS male flies influence the egg-laying response of female flies to *Pachycrepoideus* wasps:
    i. Establish two groups – one with CS males (♂presence) and another without CS male flies (♂ absence) and follow steps 11 to 17.
    ii. For ♂ presence group, set up the unexposed control (five CS females and two CS males) (step 12b) and the wasp-exposed (five CS females, two CS males, and five PP wasps) (step 12a) vials using the above-mentioned steps.
    iii. For ♂ absence group, set up the unexposed controls with five mated CS females (in step 12b) and the wasp-exposed with five mated CS females and five PP wasps (in step 12a).
      **CRITICAL:** Ensure that age-matched, mated CS females are used to set up both cohorts.
  c. To study the influence of mating on CS egg-laying response to *Pachycrepoideus* wasps (Scheme 2):
    i. Discard all the CS F1 progeny eclosed in the culture bottles into a morgue (from step 9 of preparation of *Drosophila* cultures for behavioral assay).
    ii. After 3–4 h, use CO_2_ to anesthetize newly eclosed flies and select unmated CS females on a fly pad. Transfer them to a fresh food bottle/vial.
      ***Note:*** Unmated newly eclosed females are larger than mated, older females and have a lighter body pigmentation and meconium (a dark greenish spot) on the ventral side of the abdomen. Newly eclosed females are typically enriched in the early morning hours of their light/dark cycle, although they will continue to eclose throughout the day.
    iii. If necessary, repeat the above step after a few hours to collect more unmated females.
    iv. Age these unmated flies for 6 days in the incubator before setting up the egg-laying assay with or without the parasitoid wasp (refer to steps 12 to 17).
      **CRITICAL:** Before setting up the behavioral assay, carefully examine the culture bottles/vials used to house unmated females to ensure no larval activity. Presence of larvae indicates that some flies successfully mated before they were collected and separated from males. Discard cultures with larvae.

19. The above-described steps are used to include additional testing groups, as reported in Sadanandappa et al.^1^
  a. To assess the egg-laying responses of CS flies to *Trichopria* sp. (Trical) parasitoids, which belong to the Diapriidae family, culture the wasps on CS pupae using the protocol described previously,^3^ and set up the behavioral assays using five mated female Trical wasps (7-day-old) (steps 11a and 12a) .
  b. To assess the egg-laying responses of CS flies to male *Pachycrepoideus* wasps, use five male *Pachycrepoideus* wasps (7-day-old) instead of female wasps (in step 12a.iii) to set up the PP-exposed group.
20. Note the following instructions for testing the impact of different factors such as age, mating status, cohabitation period, etc., on the egg-laying response of CS flies to *Pachycrepoideus* wasps, as reported in Sadanandappa et al.^1^
  a. To examine the egg-laying responses of aged CS flies to *Pachycrepoideus* wasps (Scheme 1):
    i. Collect newly emerged F1 CS progenies (0–12 h) from culture bottles and maintain them in the incubator as outlined in step 11b.
    ii. Regularly transfer F1 flies to a fresh bottle every 4–5 days to prevent them from getting stuck to damp media and dying.
    iii. After 20 days of aging, use these CS female and male flies to conduct the egg-laying assay, with or without the *Pachycrepoideus* wasps following steps 12 to 18.
      ***Note:*** Aged CS females (P20 - P25) lay fewer eggs than younger flies (P6 - P11).

**Scheme 2:**
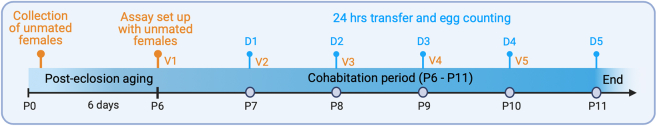
The workflow to study the influence of mating on CS egg-laying responses On post-eclosion day 0 (P0), unmated newly eclosed female CS flies collected and aged 6 days before exposing to the parasitoid wasps (P6). Every 24 h, all insects were transferred to a fresh vial (V1 to V5) for five days (D1 to D5), and the number of eggs laid was recorded.
  d. To study the effect of *Pachycrepoideus* cohabitation duration on CS egg-laying response (Scheme 3):
    i. Set up unexposed control and PP-exposed vials using the steps described in 11–13.
      ***Note:*** Age-matched flies and parasitoids should be used to set up at least 25 vials for the PP-exposed group.
    ii. After 24 h of egg-laying, transfer only CS flies from five PP-exposed vials (V1) to fresh vials (V2) and continue the egg-laying assay from D2 to D5 without wasp (steps 14 to 16). At the same time, normally transfer the rest of the PP-exposed vials and the unexposed controls (step 14). Document the egg-lay count from all vials (D1).
      **CRITICAL:** Use the negative geotaxis behavior of CS flies with gentle tapping to remove the wasp from the PP-exposed vials without CO_2_ exposure.
    iii. Repeat the above step in the following three transfers to remove wasps from PP-exposed vials for two (V2 to V3), three (V3 to V4), and four-day (V4 to V5) cohabitation of CS flies with female *Pachycrepoideus* and continue the assay to document the egg-lay for all five days.

**Scheme 3:**
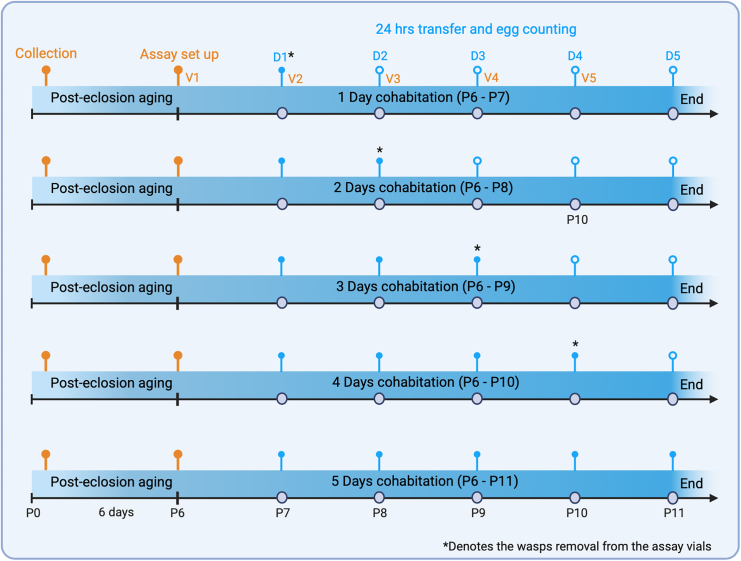
The workflow to study the effect of wasp cohabitation duration on CS egg-laying responses On post-eclosion day 6 (P6), flies cohabitated with the parasitoid wasps. Every 24 h, flies were transferred to a fresh vial (V1 to V5) for five days (D1 to D5), and the number of eggs laid was recorded. Wasps were removed before the transferring to a fresh vial depending on the cohabitation period (marked ∗).
  e. To study the effect of parasitoid withdrawal on CS egg-laying response to *Pachycrepoideus* wasps (Scheme 4):
    i. Set up unexposed control and PP-exposed vials as described in steps 11 to 16.
    ii. After 5 days of co-habitation with CS flies and female *Pachycrepoideus*, remove the wasps from all PP-exposed vials.
    iii. Continue 24 h transfers and record egg-lay for the following 6 days (D6 to D11) from the unexposed controls and PP-withdrawn cohorts.

**Scheme 4:**
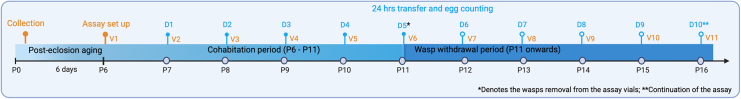
The workflow to study the effect of parasitoid withdrawal on CS egg-laying responses On post-eclosion day 6 (P6), flies cohabitated with the parasitoid wasps (P6 - P11). Every 24 h, all insects were transferred to a fresh vial (V1 to V5) for five days (D1 to D5), and the number of eggs laid was recorded. After 5 days of exposure, the wasps were removed on P11 (marked ∗) the assay is continued to record the egg-lay.
  f. To investigate the influence of parasitoid age^4^ on CS egg-laying response to *Pachycrepoideus* wasps:
    i. Collect newly emerged F1 *Pachycrepoideus* progenies from wasp-infected vials and maintain wasp culture vials as outlined in step 11a.
    ii. While aging the wasps, regularly transfer them to a fresh culture vial every 4–5 days to prevent them from dying from the deteriorating medium.
    iii. Set up separate PP-exposure groups with younger (4-day-old) and older wasps (10-day-old) along with the unexposed controls (step 12). Then, perform steps 13 to 17 to document the five-day CS egg-lay.

### Eclosion assay

**Timing: 17–18 days**

***Note:*** The eclosion assay is an optional step used to examine the influence of the parasitoid wasp on the fertilization and development of CS flies.

21. After recording the egg count, place all the vials (V1 to V5) from the above behavioral assay in the incubator.
22. At 25°C, the CS progeny (F2 flies) will start to emerge approximately nine days after the eggs are laid.
23. Use CO_2_ to anesthetize the F2 progeny before documenting the total number of male and female flies emerging from each vial for eight days from the beginning of eclosion.
24. Import the data to Microsoft Excel or GraphPad Prism for data analysis and visualization.

## Expected outcomes

*Drosophila* parasitoid wasps, which parasitize *Drosophila* larval and pupal stages,^5^^,^^6^^,^^7^ trigger various behavioral and physiological responses in fruit fly larvae^8^^,^^9^^,^^10^^,^^11^ and adults.^2^^,^^4^^,^^12^^,^^13^^,^^14^^,^^15^^,^^16^ Our protocol involves *D. melanogaster* adults and *Pachycrepoideus*, a pupal parasitoid wasp, to investigate how the presence of the wasps influences the reproductive responses of the fruit flies.^1^ We expect to observe an increase in the average eggs laid by the fruit flies after five days of cohabitation with *Pachycrepoideus* wasps compared to the unexposed controls (Figures 3A–3C). The group exposed to *Pachycrepoideus* wasps consistently shows a higher percentage of offspring eclosion (Figures 3D and 3E). However, as reported in Sadanandappa et al.,^1^ prolonged cohabitation female *Pachycrepoideus* parasitoids, but not the male wasps, leads to increased egg-laying in mated CS females and has a marginal effect on unmated flies. Various factors, including age, mating, and cohabitation duration, impact the reproductive behavioral outcomes and germline development of the fruit flies.^1^ This behavioral assay serves as a method for characterizing the circuit and signaling mechanism that drives reproductive modifications in *Drosophila* in response to *Pachycrepoideus*-specific cues and can also be used to study the interactions between host-parasitoids across different species.

***Note:*** Both the unexposed controls and the PP-exposed group show a decline in egg-laying over the five-days assay period. This gradual decrease in egg-laying reflects the aging of the *Drosophila*.

## Quantification and statistical analysis

After recording the number of eggs laid across different days (D1 to D5).

• Use Microsoft Excel or GraphPad Prism to calculate the average number of eggs laid by unexposed control and PP-exposed groups and create graphs.
• Determine the mean ± standard error of the mean from at least three independent experiments.
• Calculate the egg-lay percentage by normalizing the mean egg count of the PP-exposed group to the unexposed control egg count for each corresponding day (Figures 3B and 3C).
• Use one-way or two-way ANOVA with Tukey/Bonferroni/Dunnet’s correction for the multiple comparisons to determine the statistical significance between the unexposed control and PP-exposed groups.

For the eclosion dataset, perform the same analysis to determine the percentage eclosion rate, which is calculated by the ratio of F1 progeny to the total number of mock F1 progenies, multiplied by a hundred (Figures 3D and 3E). Also, exclude any vials with dead CS females or escapees during the transfer from the assay and the data analysis.

## Limitations

The *Pachycrepoideus* culturing and the behavioral assays outlined in this protocol are relatively simple, straightforward, and easily adaptable without special equipment or reagents. For example, the protocol described can be used to culture other parasitoid species^3^^,^^17^ and investigate the interaction of different host-parasitoid species.^4^ Thus, it is a powerful and versatile assay for research and teaching laboratories.

However, it is important to note that these assays are laborious and time-consuming, requiring careful planning and execution. Several factors, such as insect handling and maintenance, genetic background, and culture conditions, including fly medium, temperature, humidity, L/D cycles, and crowding, significantly impact behavioral outcomes. Therefore, throughout this protocol, we emphasize the importance of using age-matched, mated, healthy insects for setting up behavioral assays, limiting prolonged CO_2_ exposures, gentle insect handling to avoid mechanical stress, and following good laboratory practices for culturing wasp and fruit flies.

## Troubleshooting

### Problem 1: Parasitization efficiency

The reduced emergence of *Pachycrepoideus* parasitoids from the wasp-infected vials is related to steps 7 and 8.

### Potential solution

To maximize the parasitoid emergence from the host vial, the following factors need to be considered.

• Preparation of host cultures: Use an appropriate number of host eggs without overcrowding to synchronize the host developmental stages. Early pupal stages are more susceptible to parasitization due to reduced immune resistance. Therefore, introducing *Pachycrepoideus* adults when the *D. virilis* larvae initiate the pupation is crucial for efficient parasitization.
• Handling of wasps: Ensure gentle handling of wasps, limit exposure to high concentrations of CO_2_, make sure the wasps are not sticking to wet media after transfer, and supplement the plugs with a sufficient honey-water mixture to reduce the lethality of adult wasps. If necessary, add additional adult wasps a few days post-infection.
• Note that the host pupae that successfully mount the immune response against *Pachycrepoideus* infection kill the developing wasp egg, and adult fruit flies eclose from the pupal case before the emergence of adult wasps. It is important to remove these escaped flies to avoid *D.virilis* breeding within the host vial.

### Problem 2: Variability in the egg-lay

*Drosophila* egg-lay is regulated by both intrinsic and extrinsic factors, leading to variations in the number of eggs laid within and across different batches (steps 11 to 18).

### Potential solution

To minimize the impact of these factors on egg-laying, it is important to adhere to good fly-pushing practices as outlined in the protocol for maintaining and handling insect cultures in the laboratory. Additionally.

• Use age and genotype-matched CS flies from the healthy culture bottles maintained in the incubator under controlled conditions.
• Reduce exposure to high concentrations of CO_2_ anesthesia and avoid excessive mechanical stress during transfers. Except for the cohabitation period, fruit flies and wasp stocks should be kept and handled separately.
• Perform independent experiments on different days with at least five sets per group, ideally maintaining consistent conditions across experiments. This includes setting up the assay in the morning, conducting transfers, and counting eggs at similar times. Consider discontinuing the assay if there is a significant variability across the sets or a noticeable decrease in eggs in the experimental vials.

### Problem 3: Insect loss during the assay

Apart from insects dying in the assay vials, they will likely escape during transfer between vials without anesthesia.

### Potential solution

• Use low CO_2_ pressure during assay setup to reduce excessive anesthesia exposure and minimize the mechanical stress during the transfers to avoid insect fatalities.
• Handle the insects carefully to prevent escapes. Before placing them in the assay vials, ensure they are dry and maintained at RT without any condensed water.
• Remove any vials containing dead or escaped CS flies from the assay and exclude the data from those vials from the analysis. Replace assay vials with a dead wasp are replaced with age-matched ones during the transfers. Therefore, it is important to set up multiple sets for each condition.

## Resource availability

### Lead contact

Further information and requests for resources should be directed to and will be fulfilled by the lead contact, Giovanni Bosco (giovanni.bosco@dartmouth.edu).

### Technical contact

Technical questions on executing this protocol should be directed to and will be answered by the technical contact, Madhumala K. Sadanandappa (Madhumala.K.Sadanandappa@hitchcock.org).

### Materials availability

Fly lines and wasp stocks are available through the lead contact.

### Data and code availability

• Original data for figures in the paper are available in Sadanandappa et al.^1^
• Any additional information on the data is available from the lead contact upon request.
• This study did not generate code.

## Acknowledgments

We thank Todd Schlenke for providing the *D. virilis* and the wasps stocks and Victoria L. Marlar and Shivaprasad H. Sathyanarayana for technical assistance. This project was supported by the Human Frontier Science Program Long-Term Fellowship LT000933/2017 to M.K.S. and the National Institutes of Health Pioneer grant 1DP1MH110234 to G.B. BioRender.com was used for making the graphic.

## Author contributions

Conceptualization, methodology, and funding acquisition, M.K.S. and G.B.; investigation and writing – original draft, M.K.S.; writing – review and editing, M.K.S. and G.B.

## Declaration of interests

The authors declare no competing interests.
